# Prevalence of alveolar bone loss in healthy children treated at private pediatric dentistry clinics

**DOI:** 10.1590/S1678-77572010000300016

**Published:** 2010

**Authors:** Maria do Carmo Machado GUIMARÃES, Valéria Martins de ARAÚJO, Márcia Raquel AVENA, Daniel Rocha da Silva DUARTE, Francisco Valter FREITAS

**Affiliations:** 1 DDS, MSc, PhD, Adjunct Professor of Periodontics, Periodontics Division, University of Brasilia, Brasília, DF, Brazil.; 2 DDS, MSc, Assistant Professor of Periodontics, Department of Dentistry, University of Brasilia, Brasília, DF, Brazil.; 3 DDS, Graduate student, Department of Dentistry, University of Brasilia, Brasília, DF, Brazil.

**Keywords:** Child, Radiography, Alveolar bone loss

## Abstract

**Objectives:**

The purpose of this study was to evaluate the prevalence of alveolar bone loss (BL) in healthy children treated at private pediatric dentistry clinics in Brasília, Brazil.

**Material and Methods:**

The research included 7,436 sites present in 885 radiographs from 450 children. The BL prevalence was estimated by measuring the distance from the cementoenamel junction (CEJ) to alveolar bone crest (ABC). Data were divided in groups: (I) No BL: distance from CEJ to ABC is ≤2 mm; (II) questionable BL (QBL): distance from CEJ to ABC is >2 and <3 mm; (III) definite BL (DBL): distance from CEJ to ABC ≥3 mm. Data were treated by the chi-square nonparametric test and Fisher's exact test (p<0.05).

**Results:**

Among males, 89.31% were classified in group I, 9.82% were classified in group II and 0.85% in group III. Among females, 93.05%, 6.48% and 0.46% patients were classified in Group I, II and III, respectively. The differences between genders were not statistically significant (Chi-square test, p = 0.375). Group composition according to patients’ age showed that 91.11% of individuals were classified as group I, 8.22% in group II and 0.67% in group III. The differences among the age ranges were not statistically significant (Chi-square test, p = 0.418). The mesial and distal sites showed a higher prevalence of BL in the jaw, QBL (89.80%) and DBL (79.40%), and no significant difference was observed in the distribution of QBL (Fisher’s exact test p = 0.311) and DBL (Fisher’s exact test p = 0.672) in the dental arches. The distal sites exhibited higher prevalence of both QBL (77.56%) and DBL (58.82%).

**Conclusions:**

The periodontal status of children should never be underestimated because BL occurs even in healthy populations, although in a lower frequency.

## INTRODUCTION

For many years, periodontal disease was condition may be related to systemic diseases; and regarded as a progressive condition, affecting most (3) patients, families, or population at risk may be of the population^25^. After further investigations, it identified and included in specific prevention or has been suggested that the disease is a far more treatment programs. specific entity with only a small minority of the A number of previous studies have investigated population being susceptible to disease at a young the incidence and prevalence of periodontal age or to aggressive bone loss (BL)^8^.

Dental clinicians must diagnosis and manage properly periodontal diseases in children and adolescents. Bimstein^2^ (1991) stated the importance of early diagnosis and treatment of periodontal disease, having in mind that (1) incipient periodontal diseases in children may develop into advanced periodontal diseases in adults; (2) periodontal condition may be related to systemic diseases; and (3) patients, families, or population at risk may be identified and included in specific prevention or treatment programs.

A number of previous studies have investigated the incidence and prevalence of periodontal disease in children. The methods used have ranged from clinical measurements to radiographic assessments, and have included both longitudinal and cross-sectional studies. Epidemiological surveys have focused in bone destruction, assessed by radiographs, in order to access the prevalence of periodontal diseases in the studied populations. The population groups that have been the subjects of these studies have comprised both developed and developing countries, and have investigated the influence of additional factors such as education and race^5, 11, 30^. Studies have indicated that periodontal disease in the permanent dentition of adolescents is often preceded by BL in the primary dentition^28^. Destruction of bone remains is the most important criterion for assessing the severity of periodontitis and the identification of individuals' susceptibility to periodontal breakdown^21^. Bitewing radiographs are commonly taken in children for caries assessment and, in addition, these radiographs can also be used in order to observe the bone height around the first molars.

Thus, analyses of radiographs, used previously to caries analyzes, provide a good assessment of BL in children^4,26^. The radiographic signs as evidence of initial periodontal breakdown are (1) widening of the periodontal ligament space, (2) diffuseness or absence of the crest cortical plate, (3) thinning or absence of the trabeculae of the crestal alveolar, and (4) quantitative changes in the distance from the cementoenamel junction (CEJ) to the alveolar bone crest (ABC)^17^.

A previous literature review^13^ showed that the most objective criterion for the assessment of periodontal disease from radiographs is one which involves measuring the distance between the images of the CEJ and ABC. Furthermore, Pierro, et al.^24^ (2008) evaluated the reliability of methods caliper and computerized images to assess alveolar BL in primary teeth. Both methods were proven to be reliable. Nevertheless, there have been reports on the disadvantages of dental radiography as a diagnostic resource for detection of early periodontal lesions because it only reveals the interproximal aspects of the dentition^17^.

Epidemiological studies have shown that the prevalence of BL in the primary dentition varies between 0.27% and 28%^7, 14^. This variation might reflect the different prevalence of AP in people from different socioeconomic status, education, races or origins, and healthy condition. Particularly high prevalence of periodontitis has been reported from people of countries in Africa and Asia ^14^.

In this study, the prevalence of alveolar BL in a sample of Brazilian healthy children was assessed by analyzing the CeJ-ABC distance in bitewing and periapical radiographs collected at three private pediatric dentistry clinics in Brasília, DF, Brazil.

## MATERIAL AND METHODS

This cross-sectional study included 450 Brazilian healthy children aged 2 to 11 years (52% male, 48% female). Bitewing and periapical radiographs were collected from the patients’ dental records at three private pediatric dentistry clinics in Brasilia, DF, Brazil. ethical approval was obtained from the Research ethics Committee of the Healthy College of the University of Brasilia, Brazil.

Two examiners measured the distance from CEJ to ABC in 7,436 sites in 885 radiographs, at mesial and distal aspects of anterior and posterior teeth. A transparent ruler, graduated in millimeters, a magnifying glass and an x-ray viewer were used during evaluation. When more than one set of radiographs were present in the same record, the most recent data were chosen for examination.

Inter-and intraexaminer calibrations were performed to guarantee research reproducibility. For both calibrations, 50 radiographs randomly chosen from the total of 885 radiographs, were examined by each examiner according to the study methodology at 2-day intervals during 10 days until homogeneous results were obtained. The results of both examiners at the 10^th^ day showed 97% of agreement.

The radiographs selected had minimal or no distortion, no overlapping between adjacent tooth surfaces and good contrast, in order to provide a clear image of ABC and CEJ. Radiographs of children under orthodontic treatment and radiographs in which ABC was near carious lesions, exfoliating or erupting teeth, or teeth with inadequate restorations, endodontic treatment or trauma, were not included in the study. Tooth was considered to be exfoliating if the root surfaces had advanced to the extent that the radiographic image of the periodontal ligament was not discernible. A permanent tooth was considered to be erupting if its cusp tips had not reached occlusion in the radiograph^18^.

Data were divided in groups following the criterion adopted by Bimstein, et al.^4^ (1994): Group I- No BL: the distance from the CEJ to ABC is ≤2 mm; Group II- Questionable BL (QBL): the distance from the CEJ to ABC is >2 and <3 mm; and Group III- Definite BL (DBL): the distance from CEJ to ABC is ≥3 mm.

Statistical analyses were performed using SigmaStat software for Windows, version 3.11 (Systat Software, Inc, Chicago, IL, US). BL according to gender and group composition according to patients’ age were analyzed by the chi-square nonparametric test at 0.05 significance level. The effect of BL in teeth surface, located in the opposite side of maxilla or jaw hemi arches, was treated by Fisher’s exact test at 0.05 significance level.

## RESULTS

The patient sample was composed of 234 (52%) boys and 216 (48%) girls with age ranging from 2 to 11 years-old. Following exclusion and inclusion criteria, 885 periapical and bitewing radiographs from the 450 children supplied 7,436 sites for evaluation (Table 1).

**Table 1:** Number of bitewing and periapical radiographs evaluated in the study according to subjects' age

**Age**	**Radiographs**		**Total**
	**Bite-wing**	**Periapical**	
			
2	-	13	13
3	8	25	33
4	37	47	84
5	66	29	95
6	104	11	115
7	157	9	166
8	134	4	138
9	126	1	127
10	87	3	90
11	24	-	24
Total	743	142	885
%	83.95%	16.05%	100%

Among males, 89.31% patients were classified in Group I, 9.82% in Group II and 0.85% in group III. Among females, 93.05%, 6.48% and 0.46% patients were classified in Group I, II and III, respectively (Table 2). The differences between genders were not statistically significant (chi-square test, p = 0.375).

**Table 2:** Bone Loss (BL) according to gender

**Gender**		**Group**	
	**Group I (No BL)**	**Group II (QBL)**	**Group III (DBL)**
Male	209 (89.31)	23 (9.82)	2 (0.85)
Female	201 (93.05)	14 (6.48)	1 (0.46)

X^2^=1.962. p= 0.375. Results are presented as "number of individuals (%)". Q= questionable; D= definitive

Table 3 shows the distribution of age groups with intervals of 2 to 4, 5 to 6, 7 to 8, and 9 to 11 years in the different categories of BL. In all age ranges, absence of BL (Group I) had the highest prevalence, totaling 91.11% of individuals. The prevalence of radiographic BL in the studied population was 8.88% (QBL – 8.22%; DBL – 0.67%). According to the chi-square test, the different age ranges showed no statistically significant difference (p=0.418).

**Table 3:** Group composition according to patients' age ranges related to Bone Loss (BL)

**Age range**		**Group**		**Total number of individuals**
	**Group I (No BL)**	** Group II (QBL)**	**Group III (DBL)**	
				
2-4 years	92 (92.0)	7(7.0)	1 (1.0)	100
5-6 years	93 (87.7)	13 (12.2)	0 (0)	106
7-8 years	124 (94.6)	6(4.5)	1 (0.7)	131
9-11 years	101 (89.3)	11 (9.7)	1 (0.7)	113
Total	410 (91.11)	37 (8.22)	3 (0.67)	450

X^2^= 6.047. p= 0.418. Results are presented as "number of individuals (%)". Q= questionable; D= definitive

The number and distribution of questionable BL (QBL) sites are presented in Table 4, and the number and distribution of definitive BL (DBL) sites are presented in Table 5. The mesial and distal sites showed no statistically significant difference in the distribution of QBL (Fisher’s exact test p=0.311) and DBL (Fisher’s exact test p=0.672) in the maxillary and mandibular arches. Both arches showed a higher prevalence of BL in the jaw, QBL (89.80%) and DBL (79.40%). The distal exhibited higher prevalence of both QBL (77.56%) and DBL (58.82%).

**Table 4:** Number and distribution of Questionable Bone Loss (QBL) sites

**QBL (%)**		
	**Maxilla (89.80%)**	**Mandible (10.20%)**
		
Mesial (22.4%)	9(81.8)	2 (18.1)
Distal (77.56%)	35 (92.1)	3 (7.8)

Fisher's Exact Test (p = 0.311). Results are presented as "number of individuals (%)".

**Table 5:** Number and distribution of Definitive Bone Loss (DBL) sites

**DBL (%)**		
	**Maxilla (89.80%)**	**Mandible (10.20%)**
		
Mesial (41.20%)	12 (85.7)	2 (14.2)
Distal (58.82%)	15 (75.0)	5 (25.0)

Fisher's Exact Test (p = 0.672). Results are presented as "number of individuals (%)".

Primary dentition presented 81 sites with BL (QBL= 48; DBL= 33) and permanent dentition presented 2 sites (QBL= 1; DBL= 1). In the 83 sites with BL (1.11% of the total of examines sites), 71 (85.54%) were in the maxilla and 12 (14.45%) in the mandible. Distal and mesial surfaces had 58 (70%) and 25 (30%) sites with BL, respectively. The primary maxillary right canine presented the highest percentage of BL sites (QBL: 14.28%; DBL: 29.41%).

## DISCUSSION

Although the correct diagnosis of periodontitis requires the concurrence of bleeding on probing and loss of periodontal support, epidemiological studies have focused in the accumulative destructive effect of the disease revealed by clinical measurements of loss attachment or radiographic measurements of loss of marginal bone^12^.

Bitewing radiographic studies tend to underestimate periodontitis, because of the amount of demineralization required for lesions to show on a radiographic film. There is no agreement on what actually constitutes radiographic signs of disease due to the different opinions about the optimal position of the alveolar crest. Some authors consider BL as CeJ-ABC distance greater than 2 mm and others believe that it should be greater than 3 mm^11^.

This cross-sectional study assessed BL measuring the distance from CeJ to ABC in bitewing and periapical radiographs. In order to avoid false positive results due to the inherent limitations of radiographic assessment and in order to improve accuracy, the subjects with BL were classified as having either QBL (CeJ-ABC distance >2 mm and <3 mm) or DBL (CEJ-ABC distance ≥3 mm)^4^.

In order to avoid confounding variables and a heterogeneous sample, data was collected from healthy children and the exclusion criteria adopted eliminated factors that could contribute to periodontal destruction.

Reports on destructive periodontal disease in the primary dentition have revealed varying prevalence figures, ranging between 0.8 and 20%^3,4,18,29^. High prevalence of BL in the deciduous dentition may relate to poor oral hygiene. In the study by Matsson, et al.^18^ (1995), 28% of Vietnamese immigrant children 6 to 17 years old living with their parents in Sweden had experienced BL in their deciduous dentition, compared with 5% of a control group of Swedish children. Radiographic calculus in the primary dentition was observed in 15% of Vietnamese children, compared to 4% of Swedish children. Gjermo, et al.^7^ (1984) studied radiographic BL in 304 15-year-old Brazilian schoolchildren from a population with a low socioeconomic status. Their parameter to define BL was CEJ-ABC distance greater than 2 mm. They found BL prevalence of 28%. The present study included individuals from 2 to 11 years of age, who were grouped into the following age ranges: 2 to 4, 5 to 6, 7 to 8, and 9 to 11 years. In all age ranges, there was a higher prevalence of absence of BL, detected in 91.11% of subjects, compared to the prevalence of QBL and DBL, which were detected in 8.22% and 0.67% of the subjects, respectively. Younger children and from more advantaged socioeconomic status with easier access to information and enrolled in prevention programs are factors that may partly explain the lower prevalence of BL observed in this study compared to the sample Gjermo, et al.^7^ (1984).

Age has been reported to be a significant variable in determining the CeJ- ABC distance, witch usually increases with increasing age^26^. However, a study including only Brazilian children in the primary dentition phase, between 2 and 5 years of age showed that age had no effect on the distance CEJ-ABC^23^. Kronauer, et al.^14^ (1986) reported low prevalence, evaluating bitewing radiographs of 16-year-old schoolchildren in Switzerland. The clinical criterion was CeJ-ABC distance greater than 2 mm at interproximal areas of first molars. The research excluded children with poor oral hygiene, with heavy dental calculus accumulation and subjects with factors that could increase plaque retention. This Swiss survey found BL prevalence of 0.27%. There are several possible explanations for these differences in prevalence, including ages of individuals assessed, exfoliating or erupting tooth, oral hygiene, caries, restorations, variations in radiographic technique and in the number of surfaces scheduled for examination, and the sample selection method^5,12^.

The prevalence of radiographic BL in the present study was 8.88% (QBL – 8.22%; DBL – 0.66%). The exclusion criteria adopted and the studied sample, composed of healthy subjects from private clinics contributed to the low DBL prevalence observed.

The present study found BL more frequent in the maxilla (85.45%) than in the mandible (14.55%). Other studies found the same differences. Bimsten, et al.^3^ (1988) reported that 73% of the affected surfaces were located at maxilla. Shapira, et al.^26^ (1995) found CEJ-ABC distances in the maxilla greater than in the mandible (p=0.0001). Other studies^7,9,13,20^ also found BL more prevalent in maxilla. Shapira,et al.^26^ (1995) suggested that this difference was due to the different growth pattern or bone composition of the maxilla when compared to the mandible.

In this study, the distal surface had higher prevalence of both QBL (77.56%) and DBL (58.82%) compared to the mesial surface, which showed 22.4% and 41.20 of QBL and DBL, respectively. The eruption of the permanent first molar, especially in children at 7 years of age, has been identified as one of the factors to consider when evaluating a CEJ-ABC distance at the distal surface of the primary second molar^5^. However, since the erupting teeth were not included in the evaluation, the higher prevalence of BL in the distal aspect should be attributed to other possible factors. Hull, et al.^10^ (1975) and Nielsen, et al.^20^ (1980) found that self-administered oral hygiene at distal surfaces is more difficult than at mesial sites. These findings were also reported by Nevertheless, others studies^6,9,15^ found BL more frequent at mesial surfaces than at distal ones. Latcham, et al.^15^ (1983) reported that this might be due to the fact that mesial sites erupt into the mouth in advance of distal surfaces, and thus are exposed to destructive etiological factors for a longer period.

The primary maxillary right canine had the highest percentage of interproximal sites with bone resorption (QBL – 14.28%; DBL – 29.41%). Shapira, et al.^26^ (1995) found the canines with the greatest CEJ-ABC distance and the second molars the smallest. In studies that evaluate only bitewing radiographs, the primary first molar had the highest BL prevalence^15,27,28^.

Boys and girls presented similar CEJ-ABC distances, without a statistically significant difference regarding the classification in groups I, II and III (Table 2). In a recent study with a sample of Brazilian children, Pierro reported no effect of gender on the distance CeJ-ABC. Other studies^16,19,26^ did not find differences in BL when comparing both genders. Papapanou, et al.^22^ (1988) found, statistically, greater BL among males than females. However, Sjödin, et al.^28^ (1993) reported a female-to-male ratio of 1.7:1 in BL groups.

The ethnical origin of the study population was not evaluated in this study. Some publications that considered this variable found higher BL prevalence in African and African-Americans and lower BL prevalence in Caucasians^1^.

## CONCLUSIONS

Within the limitations of the present study, the following conclusions can be pointed out: 1. The low prevalence of alveolar BL in the healthy children from private pediatric dentistry clinics examined in this study may have been influenced by factors such as age, oral hygiene, socioeconomic status and education; 2. In despite of some diagnostic limitations, bitewing and periapical radiographs are useful in epidemiological studies because they are daily required during clinical practice and are usually kept in the patient’ records after the treatment, providing easy management of these data; 3. In spite of the low prevalence, caution should be exercised when children are screened for alveolar BL because of the usual slow course of periodontal disease.
